# Optimal design and development of a fast steering robot inspired by scallops

**DOI:** 10.3389/fbioe.2023.1297727

**Published:** 2024-01-08

**Authors:** Yumo Wang, Tianyu Gao, Shunxiang Pang, Jiajun Xu, Xiayu Tao, Junqin Yang, Wentao Sheng

**Affiliations:** ^1^ School of Intelligent Manufacturing, Nanjing University of Science and Technology, Nanjing, China; ^2^ Department of Automation, University of Science and Technology of China, Hefei, China; ^3^ College of Mechanical and Electrical Engineering, Nanjing University of Aeronautics and Astronautics, Nanjing, China; ^4^ National Synchrotron Radiation Laboratory, University of Science and Technology of China, Hefei, China

**Keywords:** CFD, scallop robot, drag reduction, double-hole jet propulsion, steering ability

## Abstract

The improvement of the steering performance of jet robots is challenging due to single inflexible jet aperture. Scallops provide a potential solution with hard shells and a double-hole jet propulsion, which are expected to achieve fast steering movement under water. Inspired by scallops, a bionic propulsion dynamic mesh is proposed in this article, and a three-dimensional computational model of scallops is established. We further calculated the scallop propulsion mechanism under the swing of shells with different shapes. The coupling of simultaneous swing of two shells and their coupling with velum are presented, revealing the unique movement mechanism of Bivalvia. Based on this, the advantages of the double-hole jet propulsion are applied to develop a scallop robot with excellent steering capabilities. Experiments are conducted to verify the steering performance of the scallop robot.

## 1 Introduction

The current underwater bionic propulsion mechanisms mainly include body and/or caudal fin (BCF) propulsion (Byun et al., 2011; Chowdhury et al., 2011; Zhang et al., 2016; Scaradozzi et al., 2017), middle and/or paired fin (MPF) propulsion (Zhou and Low, 2011; Niu et al., 2012; Li et al., 2016), and jet propulsion (Villanueva et al., 2011; Lin and Guo, 2012; Serchi et al., 2012; Frame et al., 2018). Among the three types of propulsion, BCF and MPF have been fully studied (Wang et al., 2018). Jet propulsion can be divided into two types according to the number of jet apertures: single-jet and multi-jet aperture propulsion (Wang et al., 2020). The research on jet propulsion is still limited to squid (Anderson and Grosenbaugh, 2005; Bartol et al., 2009a; Bartol et al., 2009b; Staaf et al., 2014), jellyfish (Dabiri et al., 2006), dragonfly larvae (Mill and Pickard, 1975; Roh and Gharib, 2018), and other forms of single-jet aperture propulsion. Such creatures usually complete water absorption and water extrusion through the same jet aperture. Scientists have imitated their propulsion mechanisms to develop some bionic jet robots with limited steering capabilities and complicated structures. For example, in 2019, Yu et al. designed a barycenter adjustment mechanism to realize a novel robotic jellyfish capable of 3D motion (Yu et al., 2019). In the same year, Wang et al., 2019 designed a steerable aperture with crank connecting rod, steering gear, and other structures. In 2020, Christianson et al. designed a cephalopod-inspired robot with a fixed-angle aperture to achieve the fastest steering speed of 50°/s (Christianson et al., 2020). The multi-jet aperture propulsion is rare in the biological world, and scallop is the main representative. It adopts the form of double-shell swing, which is similar to the dual caudal-fin robotic fish, and it can form a double-hole jet propulsion with velum.

To study the motion mechanism of scallops, Le et al., 2013 simplified the scallop shell into a two-dimensional contour in 2013, studied the influence of the simplified scallop shell on the flow field pressure and vortex street, and discussed the effects of grooves in the scallop shell on the flow field. However, 3D numerical analysis of scallops is scarce. In 2014, Qiu et al., 2014 developed a miniature scallop robot that can move in a low-Reynolds-number environment. In 2019, Robertson et al. successfully developed a bionic scallop robot without steering ability (Robertson et al., 2019).

In the previous works, we developed scallop robots through the mode of double-hole jet propulsion (Wang et al., 2018; Wang et al., 2020). However, the turning speed of the robot is limited and has not been quantified, which is one of the main parameters of the robot’s steering ability and whether the robot is practical. The design of scallop robot should be optimized to achieve fast steering.

In this paper, CFD analysis of scallop is proposed to optimize the robot design. The main contributions of the article are as follows.

(1) A bionic propulsion dynamic mesh is designed and the computational models of the scallop shells are constructed to optimally design the shell structure of the scallop robot.
(2) The pressure distribution and the join force of scallops are modeled, and the influence of jet aperture position is explored, based on which, the driving motor, artificial velum, jet apertures and steering system of the scallop robot are therefore optimized.
(3) The robot prototype and experimental platform are constructed. Experimental results show that the robot can achieve fast linear motion and steering compared with current high-mobility jet robots.

The remainder of this paper is organized as follows. Section 2 introduces the three-dimensional (3D) calculation results for the swing motion of shells with different shapes, the coupling of simultaneous swing of two shells, and the coupling of velum and two shells are introduced in Section 3. Section 4 presents the experiments and the analysis of the results. Finally, the conclusion is provided in Section 5.

## 2 Computational fluid dynamics analysis of scallop shells

To gain intuitive insight of the fluid mechanism of scallops, the 3D unsteady computational fluid dynamics (CFD) model of the shells is established. The flapping swimming of the scallop is governed by the three-dimensional in-compressible Navier-Stokes equations. The governing equations for the fluid flow can be written in a dimensionless form as

$$\rho \frac{DV}{Dt}=F-\nabla p+\mu \nabla^{2}V$$ (1)

and

∇⋅u=0 (2)

Where ρ and μ are the density and dynamic viscosity of the fluid, $\frac{D}{Dt}$ is the total time derivative, V is the fluid vector, F is the body force acting on the fluid and p is the pressure.

### 2.1 Simulation parameters

The simulation is proceeded via fluid simulation software FLUENT. In this model, the turbulent models are set as realizable *k-ε* model. No-slip conditions are applied at the scallop surfaces and walls, whereas the outlet and inlet boundary condition are set as pressure outlet conditions and velocity inlet conditions, respectively. The semi-implicit method for pressure-linked equation (SIMPLE algorithm) is used to achieve the pressure–velocity coupling.

### 2.2 Motion model parameters

In order to analyze the thrust characteristics of different shells, the rotation axis of shells is fixed at the origin of the coordinate system. The shells only undergo rotational motion and do not undergo translational motion. To accurately simulate these motion characteristics, the motion function is set as a piecewise function. The angular velocity *ω* of the upper and lower shells is set to be the same:

$$\omega =\left\{\begin{array}{c} \omega_{1}rad/s\;0<t\leq t_{1}\\ \omega_{2}rad/s\;t_{1}<t\leq T \end{array}\right.$$ (3)

where $\omega_{1}$ is the angular velocity of the shell opening motion, $\omega_{2}$ is the angular velocity of the shell closing motion, and $t_{1}$ is the duration of opening shells. The motion function is written and compiled in Visual C++ and linked to the solver. In this computational model, the computational domain consists of 390,066 tetrahedral elements. Moreover, dynamic mesh technology is used in the mesh motion, and mesh updating methods are set as smoothing and remeshing.

**FIGURE 1:**
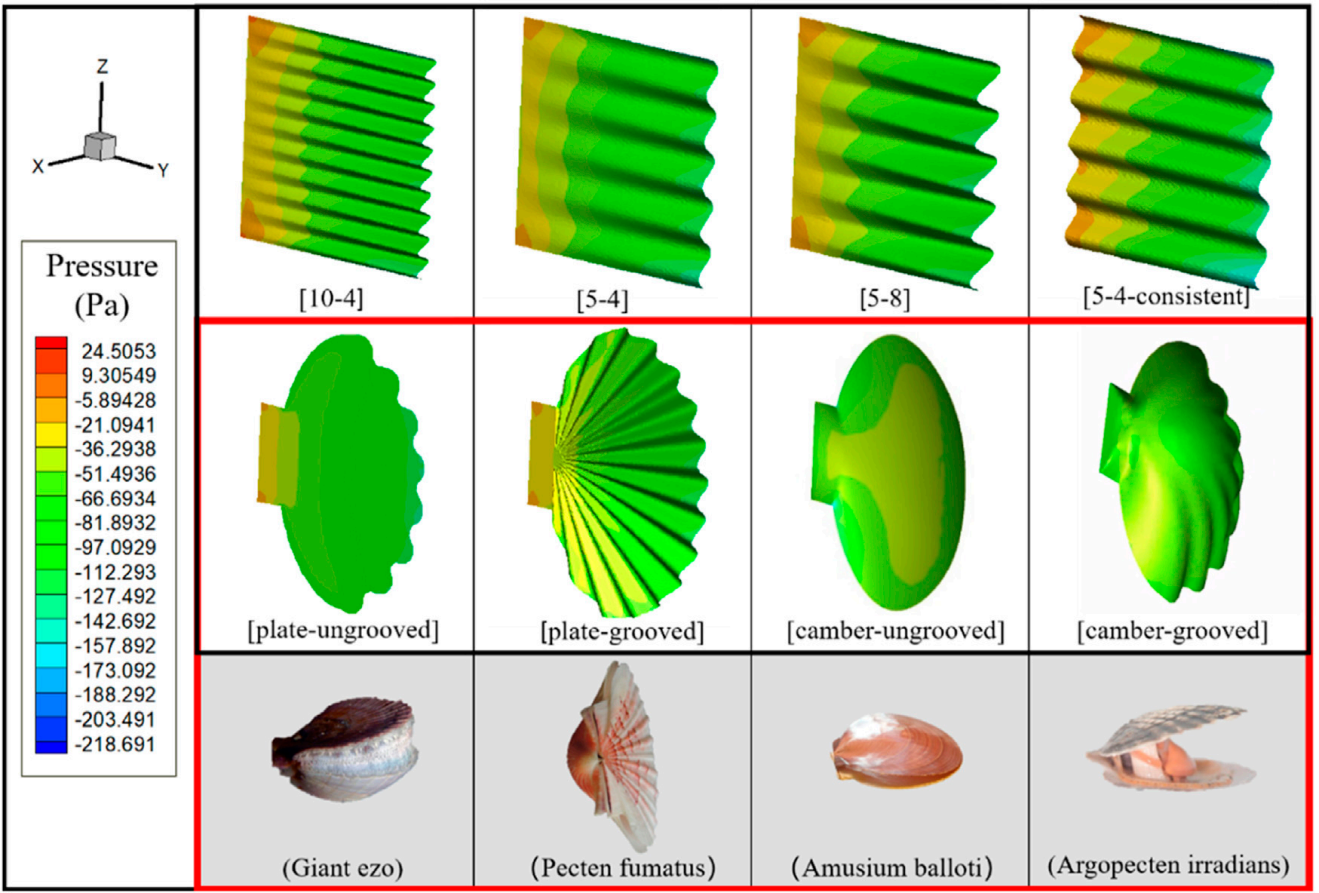
The first row represents the simulation computational model of rectangular shells. The middle row depicts four different simulated computational models of scallop shells. The bottom row shows the corresponding biological prototypes of the four scallop shell models.

The scallop shell has a radial groove structure, and the size of the groove structure decreases and tends to disappear near the swing axis. The groove structure can enhance the strength of the shell, but its effect on the resistance of the scallop movement is still unknown. In order to design the robot shell with optimal drag reduction effect, in this section, different shell models with the same projection area on the plane are designed for 3D finite element method simulation to explore the influences of different shapes, grooves, and cambers on the propulsion effects. The 3D models and corresponding biological prototypes are shown in Figure 1.

**FIGURE 2:**
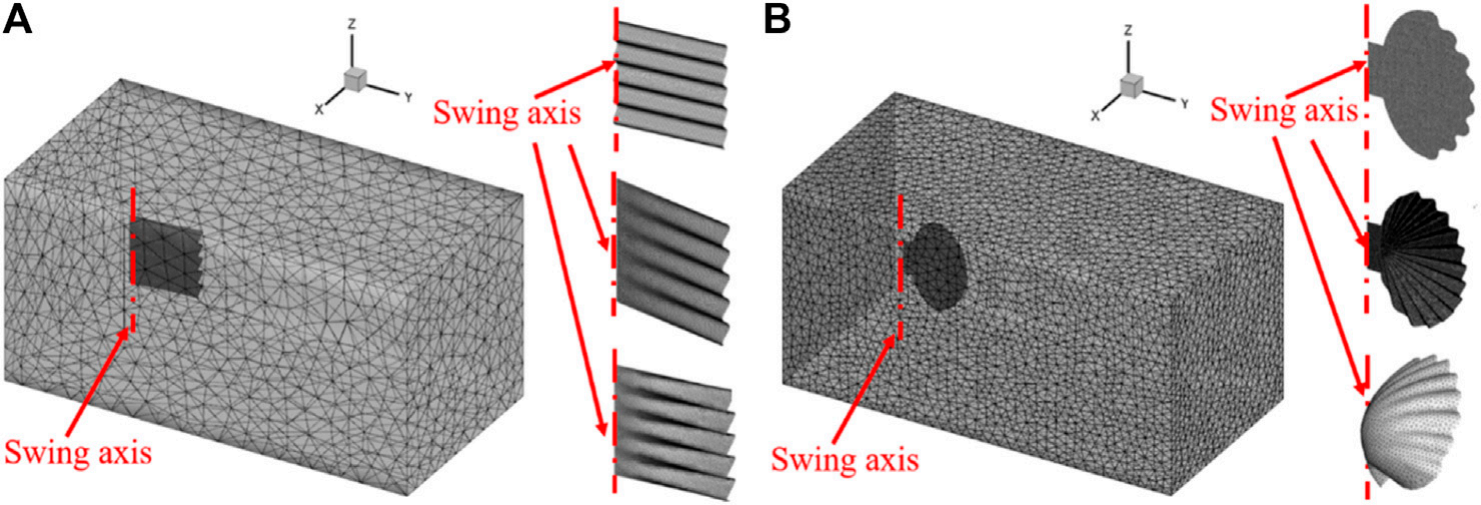
**(A)** Computational meshing of rectangular shells. **(B)** Computational meshing of scallop shells.

The naming rule of the rectangular shell is: [A-B] or [A-B-Consistent], where “A” is the number of complete waves, “B” is the wave crest height, and “Consistent” indicates that there exist grooves near the swing axis of the model. For scallop-shaped shells, the naming rule is [C-D], where “C” indicates that the shell is a plate or a camber, and “D” indicates whether it has grooves. The motion function of the scallop shells is set as the periodic swing of sin (2*PI/(T)*time), with a frequency of 2 Hz and an amplitude of 15°. We set $t_{1}=0.132$ s, T=0.33 s based on previous studies (Wang et al., 2020). The swing axis is at the far left of the model, and the value of inlet velocity is set as 0.01 m/s. Tetrahedral grid (Biswas and Strawn, 1998) is selected to capture the surface type (Frey and Alauzet, 2005), such as scallop shell; and the plane model is set to sheet mesh (Pérez and Vakkilainen, 2019). The minimum and maximum grid size is 1 mm and 10 mm, respectively. The mesh updating methods are set as smoothing and remeshing, wherein smoothing method realizes the local deformation of the mesh. The smoothing method is the spring/Laplace/boundary layer. The elasticity factor is set to 0.1, the convergence tolerance is 0.001, and the number of iterations is 20. The remeshing method is the local cell and face, which can realize the regeneration of local grid and avoid the negative volume problem in the process of calculation model movement. The specific grid generation is shown in Figure 2. The simulations are conducted in a fully submerged water environment, where the interior and exterior of the shells are filled with water.

**FIGURE 3:**
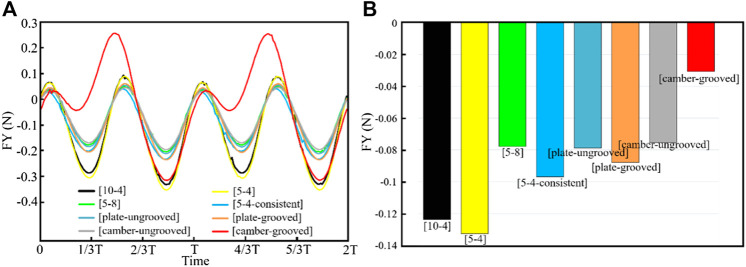
Force generated by the shells during the swing. **(A)** Instantaneous force; **(B)** Average force.

We adjust the parameters and mesh generation to calculate the change of the instantaneous force generated by different shell models in the *Y*-direction, as shown in Figure 3A. Particularly, the value of the force is obtained by superposition of the propulsion forces inside and outside the shell. From the figure, the instantaneous force generated by the camber-grooved shell is the maximum force generated along the positive direction of the *Y*-axis, and its value reaches 2.5 times of the sub-maximum value.

We further calculate the average force of each model within one cycle. As shown in Figure 3B, the propulsion effect produced by different shapes of shells during the swing process is far different. The average force on each shell is negative in the *Y*-direction. That is, the shells will produce resistance to the movement of scallops during the swing process. In the different shell models during the swing process, the camber-grooved shell produces the least resistance, which suggests that the *Argopecten irradians* have the best drag reduction effect among these four types of scallops. Besides, grooves can significantly reduce the resistance of the shell during the swing process by 59.4%. Therefore, the groove structure is recommended in the design of robot shell.

## 3 Computational fluid dynamics analysis of scallop motion process

The optimal design of individual shells has been exhibited above, in this section, we further construct a 3D model of the complete scallop with two shells for simulation. For the mesh setting of the scallop velum, we also adopt the dynamic mesh technology to achieve the mesh motion, and the mesh generation is consistent with Section 2.2. The motion function of the scallop shells is set as the periodic swing of sin (2*PI/(T)*time) which is similar to real scallops, and the incoming flow speed is set to 0.3 m/s.

**FIGURE 4:**
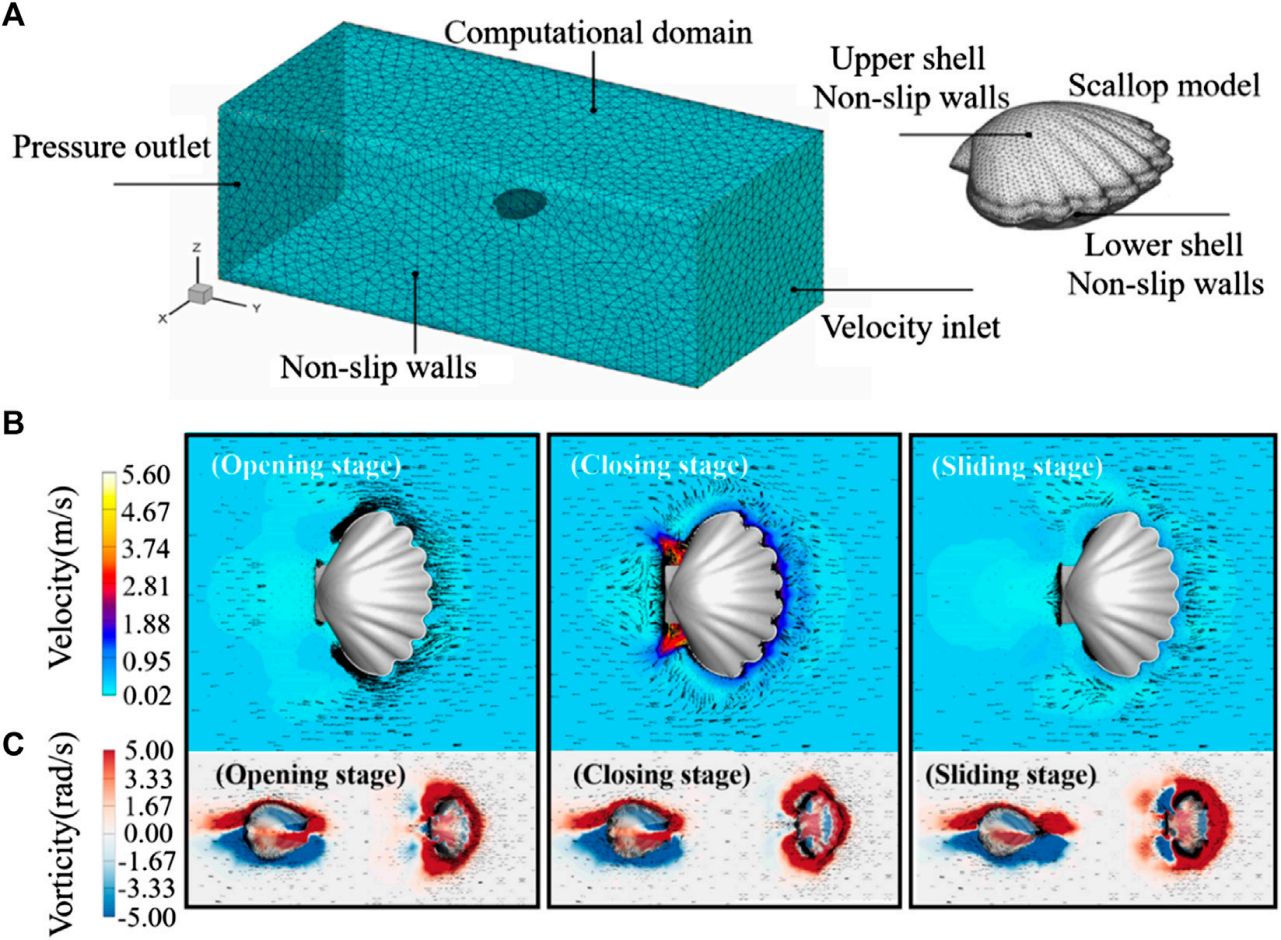
**(A)** Schematic of scallop 3D computational model. **(B)** Velocity distribution contour of the robot. **(C)** Vorticity contour of the robot. The black arrow indicates the velocity vector.

The setting of the scallop 3D calculation model and boundary conditions is shown in Figure 4A. The velocity and vorticity contours of the scallop during opening, closing, and sliding stages are shown in Figures 4B,C, respectively.

As shown in the opening stage of Figure 4B, the internal negative pressure region causes a large amount of water to be absorbed into the scallop shells from the front of the scallop. The jet apertures at the tail of the scallop are not obstructed, but there is almost no water sucked into the scallop from apertures. During the closing stage of the shells in Figure 4B, the velocity of the water flow, which is injected from the jet aperture, is extremely fast. In the sliding stage of Figure 4B, the shells of robot remain closed, and the water around the front velum side is blocked out, which leads to resistance against the movement.

From these simulation results, it can be seen that scallops mainly absorb water from the front side of the movement and eject water from two rear jet apertures. Therefore, the artificial velum should be designed with check valve function, that is, it allows water to enter the body during water opening stage and blocks the outflow of water during closing stage. Since the jet apertures do not affect the water intake during the opening stage, they can always remain open during the robot’s linear motion. To study the fluid mechanism of the scallop swimming further, Figure 4C depicts the vorticity distributions of the scallop during the opening, closing, and sliding stages. The positive vorticities are colored red, whereas the negative vorticities are colored blue; and they are attached around the upper and lower shells, which causes the shape drag. Moreover, in the jet aperture during the closing stage, there exists many positive vorticities distributed in the wake, which leads to a stronger jet flow. In addition, the vorticity of the upper and lower shells of the robot is basically symmetrical, which can ensure that the robot does not jump up and down.

**FIGURE 5:**
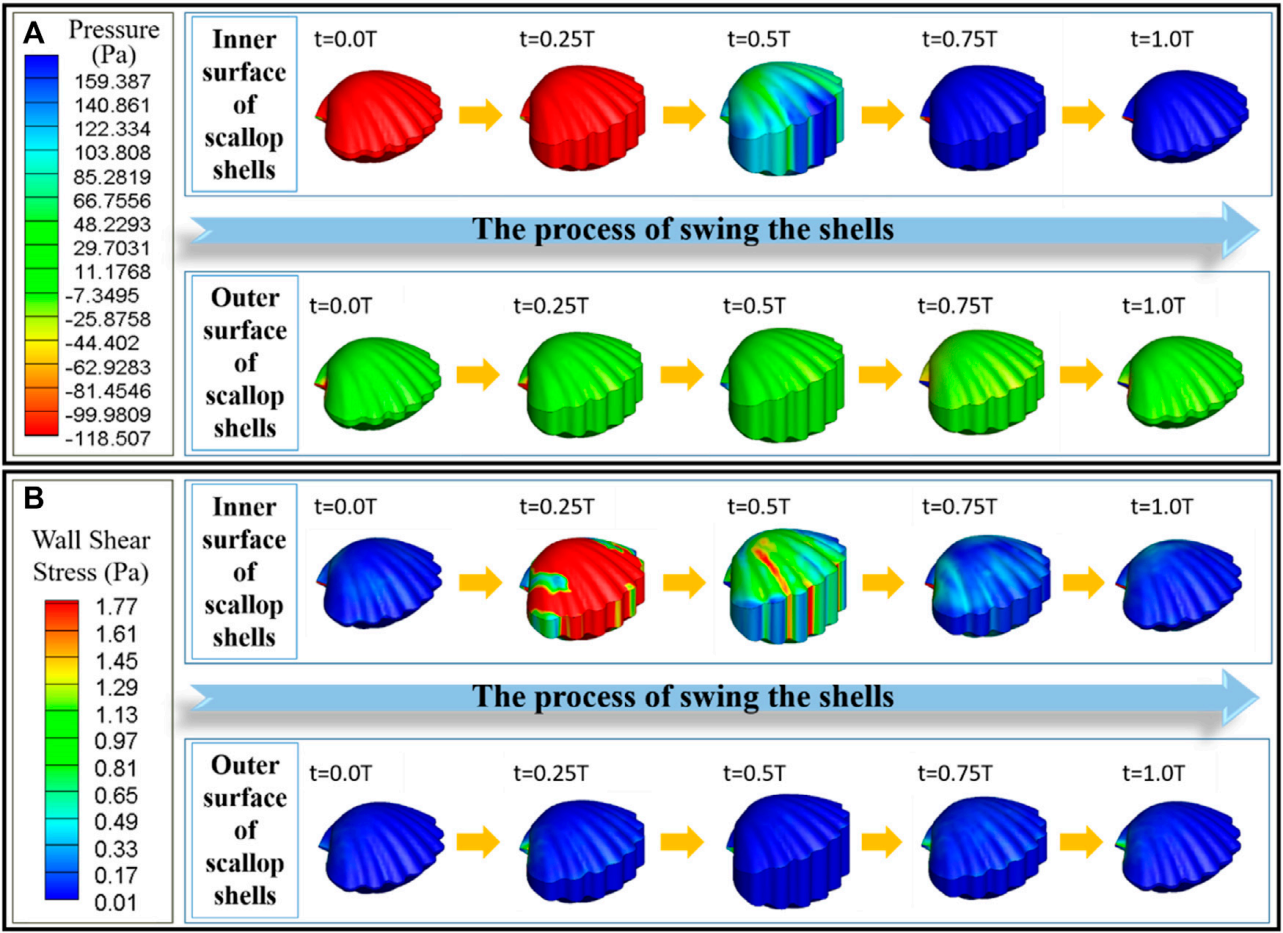
**(A)** Pressure distribution diagram of the scallop shells and the velum. **(B)** Wall shear stress distribution diagram of the scallop shells and the velum. In each diagram, the top row represents the inner surface, and the bottom row represents the outer surface.

As shown in Figure 5A, the pressure on the inner surface of the scallop is negative during the opening stage of the shells; thus, the fluid can be sucked into the cavity. In the closing stage of the shells, the pressure on its inner surface is positive, through which the fluid can be discharged from the body. The pressure of the inner surface of the velum is consistent with the pressure inside the cavity of the scallop. That is, the velum tends to bend inward under a negative pressure in the opening stage of the shells and tends to bend outward during the closing stage of the shells. In fact, when designing the artificial velum, we found that the flexible sheet is easy to bend inward and difficult to bend outward if it is fixed on the inside edge of the lower shell in a “C-shape”. Although scallops can absorb water and block flow by actively stretching and retracting the velum, we can use the unidirectional bending characteristics of the flexible sheet under pressure to construct the bionic design of the artificial velum. From Figure 5B, the wall shear stress generated on the inner surface of the scallop during the closing stage is significantly smaller than that generated during the opening stage. This facilitates an increase in the velocity of the jet flow during the ejection stage, thereby enhancing the scallop’s movement speed. Furthermore, the wall shear stress on the outer surface of the scallop is relatively low, indicating that the scallop experiences relatively low external resistance throughout the entire motion cycle.

**FIGURE 6:**
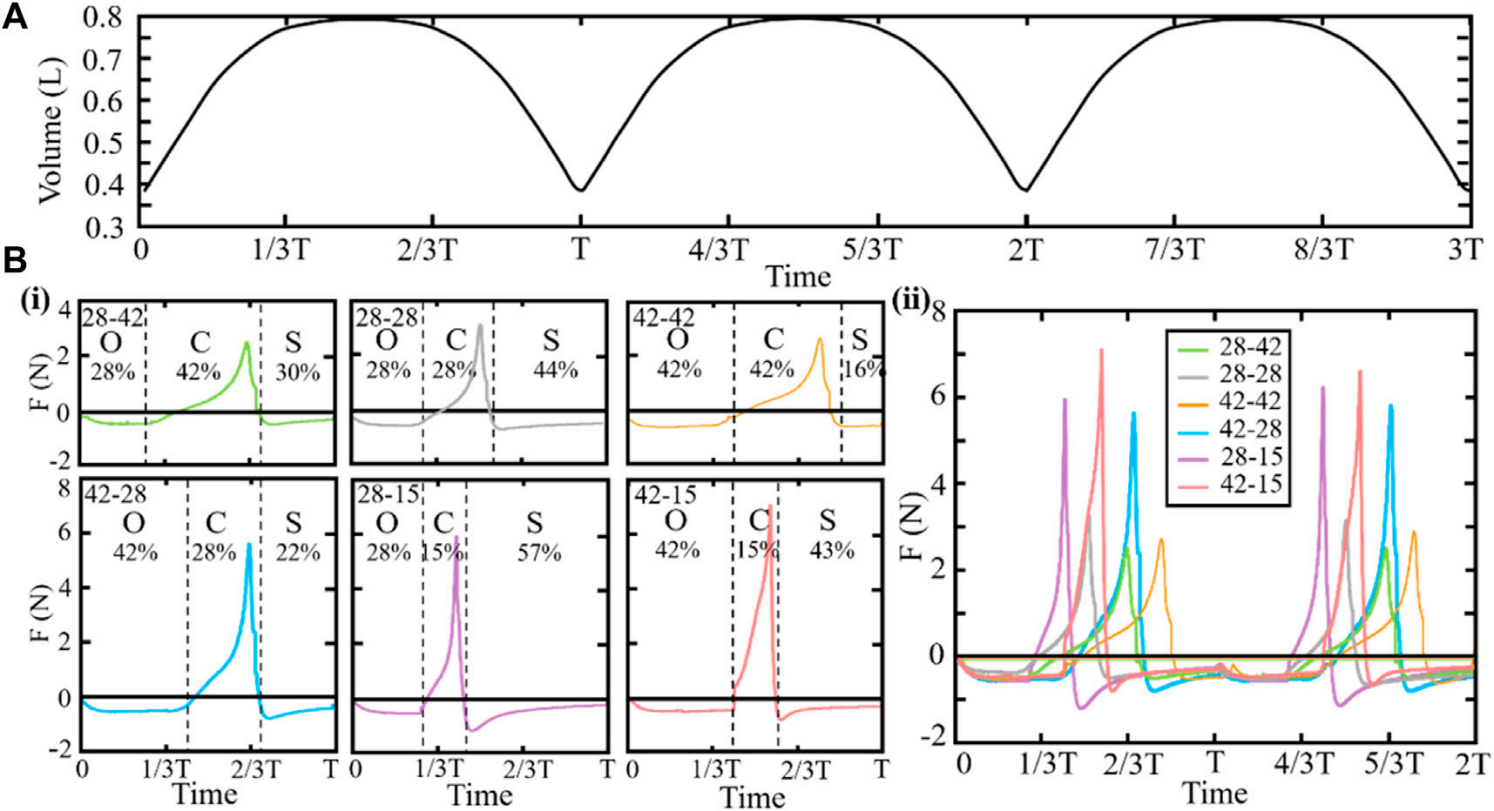
**(A)** Water content in the cavity. **(B)** Magnitude and direction of the join force of the scallops. “O,” “C,” and “S” represent the opening, closing, and sliding stages of the scallop, respectively. In naming the scallops with different ratios, the left and right numbers represent the opening and closing time ratios, respectively. (i) Individual data plots of the join forces for the scallops. (ii) Consolidated graph of the join forces for the scallops.

We further obtain the data of water content in the cavity changing with time during the process of shell swing. As shown in Figure 6A, during the opening and closing movements of the shells, the volume of the fluid that can be discharged from the cavity is almost equal to the volume of the fluid that cannot be discharged, which is determined by the shape of the shells and the initial opening angle. In addition, after the scallops absorb water and before drainage, there exists a time period when the volume of the cavity changes rarely. By adjusting the time ratio of the opening, closing, and sliding stages, the scallops can adjust the shape of the volume curve inside the cavity during the clapping process to realize the adjustment of the propulsion force.

In order to further clarify the motion process of the scallop and provide assistance for the selection of the robot’s driving motor, we have set different opening and closing ratios according to our previous studies (Wang et al., 2020). We set the different motion functions of the scallop shells and calculate the join force of scallops with different time ratios of opening, closing, and sliding stages, as shown in Figure 6B. Regardless of the ratio, the join force is positive only during the closing stage, that is, only the closing stage positively affects the scallop movement. In addition, the time cost of three stages varies, the join force is different, and the largest instantaneous join force is 7.1 N. This investigation allows us to explore how variations in these time parameters may impact scallop performance and provide insights into potential improvements in scallop biomechanics.

**FIGURE 7:**
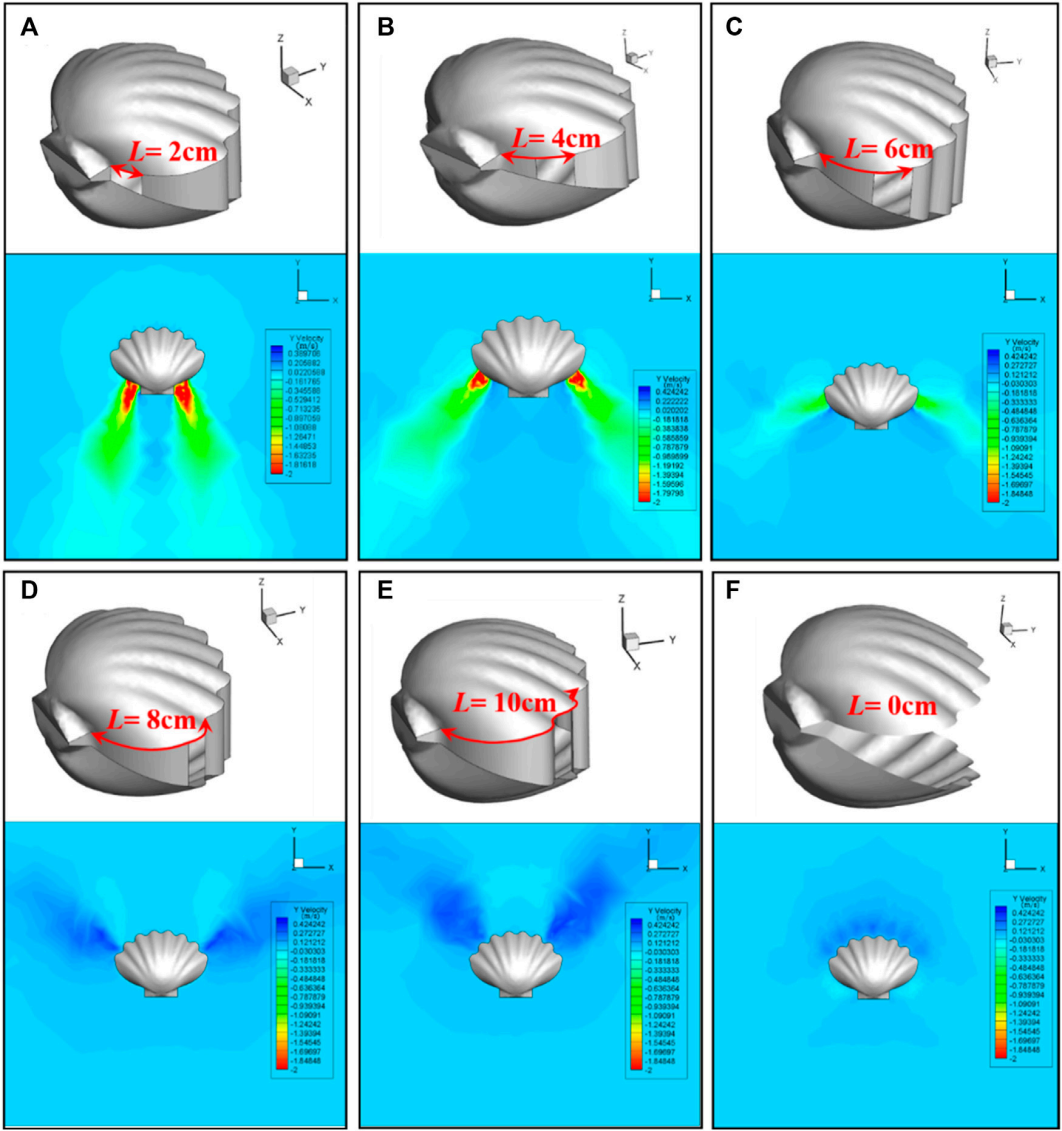
Scallop model settings and flow field simulation results for different positions of jet aperture. **(A)**, **(B)**, **(C)**, **(D)**, **(E)** represent L = 2 cm, L = 4 cm, L = 6 cm, L = 8 cm, L = 10 cm, respectively. **(F)** The control group, denoted as L = 0 cm, represents the scallop without velum.

The scallop forms the jet apertures by stretching and retracting the velum, according to which the position of the jet apertures can be adjusted, and the propulsion direction can be determined. In addition, the realization of scallop’s reverse motion and sharp stop can be regarded as a special case for scallop to adjust the position of the jet apertures. To explore the influence of aperture position on jet propulsion, scallop models with different jet apertures are established and numerical calculation results are obtained. The arc length *L* between the farthest end of the jet aperture and the posterior end of the scallop is set as the independent variable, while keeping the other parameters constant. The motion function of the scallop shells is set as sin (2*PI/(T)*time). Model setting and flow field simulation results are shown in Figure 7.

**FIGURE 8:**
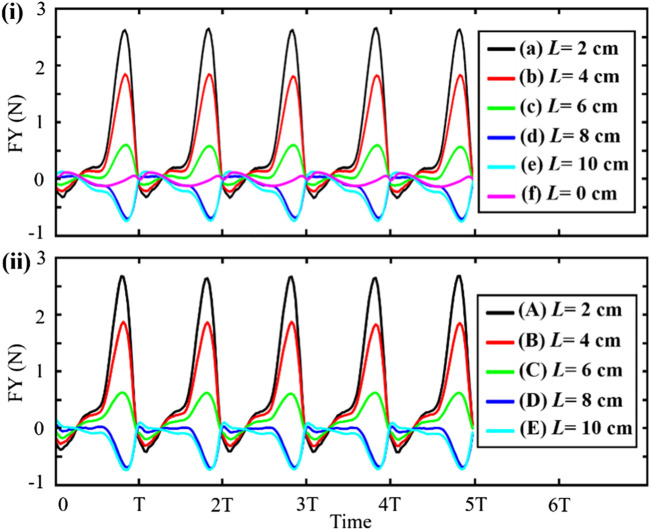
(i) Resultant force generated by the jet aperture at different positions. (ii) The force generated by the shell swing is subtracted from the resultant force.

As shown in Figure 7, the position of jet apertures has a significant influence on the jet direction, jet intensity, and accompanying flow field of the scallop. We further calculate the resultant force in the *Y*-direction generated by the jet apertures at different positions of the scallop. The calculation method of the resultant force is as follows. The internal and external forces of the two scallop shells, the internal and external forces of the velum, and the propulsion force formed by the jet are in superposition. The resultant force is shown in Figure 8i.

In Figure 8, the maximum resultant force appears in curve (a) where the jet aperture is immediately adjacent to the hinge, and the maximum resultant force is 2.7 N. This result provides a reference for the position of the robot’s jet apertures. In addition, the resultant forces produced by the scallops in Figures 8a–c are slightly different, and the positive resultant forces generated by these jet apertures are greater than that of the negative resultant forces along the *Y*-axis. The resultant forces produced by the scallops in Figures 8a–c are approximately equal, and the negative resultant forces generated by these jet apertures are greater than that of the positive resultant force. Comparing curve (f) with other curves in Figure 8i, the swing of scallop shells has far less influence than the jet propulsion on the scallop movement. By subtracting the data of curve (f) from the data of the other five curves, the effects of scallop swing shells on motion can be roughly removed, and the data of propulsion force generated by approximate pure jet can be obtained, as shown in Figure 8ii. From the figure, the propelling force of the scallop jet increases sharply and then decreases sharply after reaching the maximum peak. In conclusion, although the scallop motion is generated by the coupling of the jet and the swing of the shells, that scallops can change the jet propulsion force and motion direction by adjusting the velum without changing the swing speed of the shells.

**FIGURE 9:**
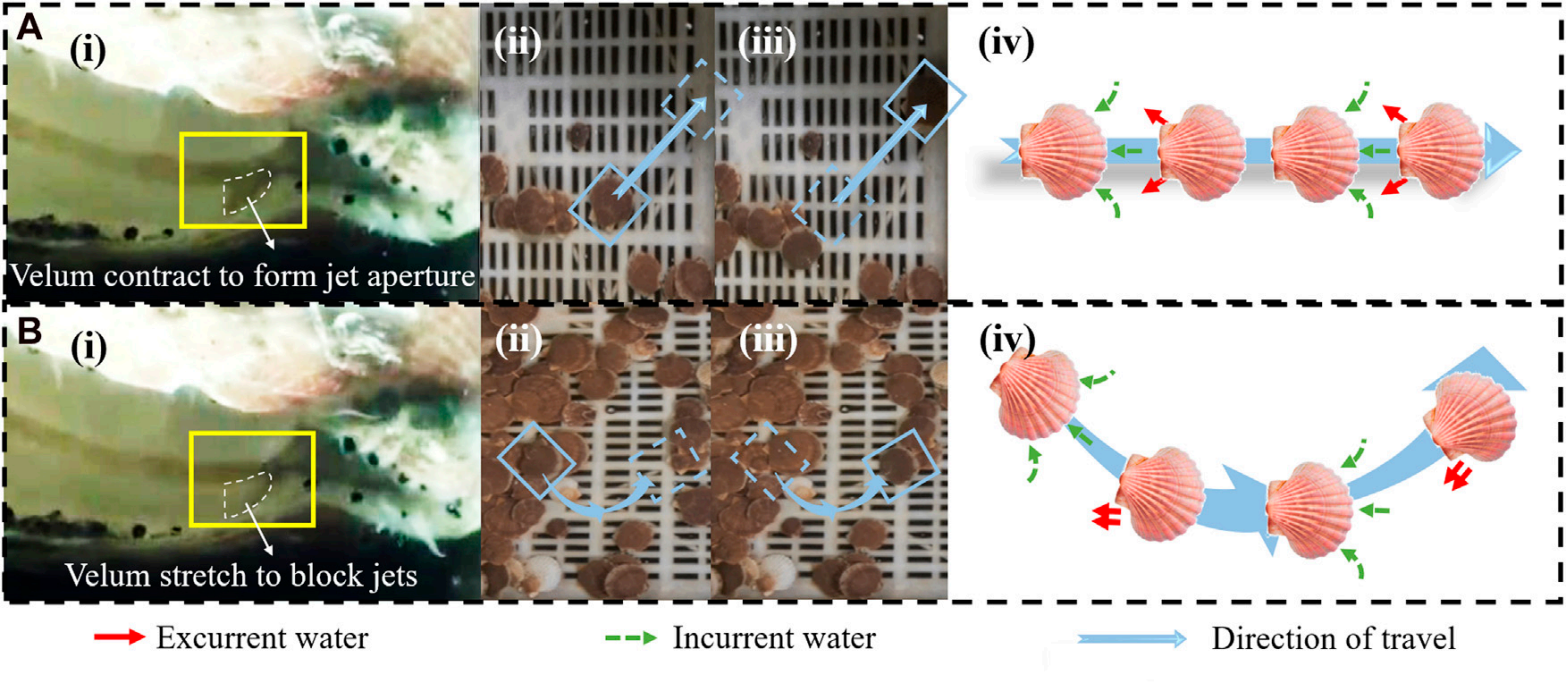
**(A)** Scallop moves forward. **(A-i)** Form symmetrical jet apertures on left and right sides. **(A-ii)** Instantaneous photo before straight-line motion of the scallop. **(A-iii)** Instantaneous photo after straight-line motion of the scallop. **(A-iv)** Schematic diagram of water suction and jetting during straight-line motion of the scallop. **(B)** Scallop turns left. **(B-i)** Left velum contract to reduce the size of the left jet aperture. **(B-ii)** Instantaneous photo before the scallop turns left. **(B-iii)** Instantaneous photo after the scallop turns left. **(B-iv)** Schematic diagram of water suction and jetting during left turn of the scallop.

When the position of the jet aperture is in case (a) in the Figure 7, the turning motion of scallop is achieved by adjusting the velum to form an asymmetric jet. When the size of the two jet apertures is different, the scallop would turn to one side where the jet aperture is smaller. The turning mechanism of the scallop is shown in Figure 9.

## 4 Robot development and experiments

### 4.1 Robot development

**FIGURE 10:**
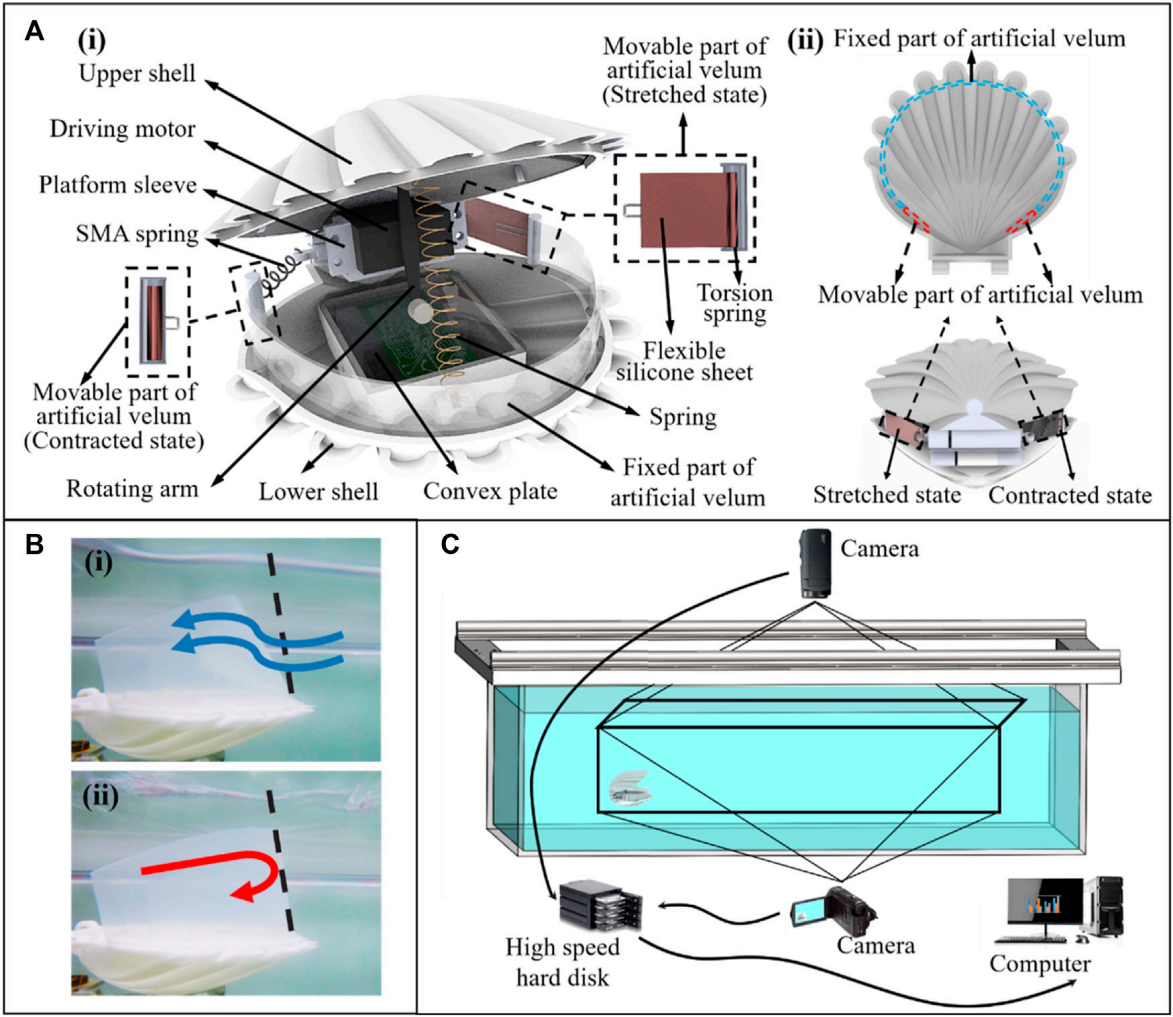
**(A-i)** Structure of the scallop robot. **(A-ii)** Top view of the artificial velum and working mechanism of the movable parts of artificial velum. **(B-i)** Principle of water flow into the cavity through the fixed part of artificial velum. **(B-ii)** The principle of water cannot flow from the fixed part of the artificial velum out of the cavity. **(C)** Experimental platform.

Based on the structural characteristics of real scallops and the simulation results mentioned above, we have developed a scallop robot with dimensions of 10 cm in length, 15 cm in width, and weighing 0.7 kg. It consists of two shells, driving unit, and artificial velum, as shown in Figure 10A. According to the simulation results of Figure 3, the shells of the scallop robot are designed in the shape of camber-grooved to achieve drag reduction. The shells are made of resin material and fabricated by 3D printing. The two shells are connected by a rotatable platform sleeve located at the rear. The driving unit is composed of a driving motor, a rotating arm, a couple of convex plates and a spring. The couple of convex plates are fixed on the inner side of the robot shells. The driving motor drives the rotating arm to contact the convex plates and open the shells. After the shells are opened to the maximum, two shells are pulled and closed by the spring. The power of the driving motor is determined according to the simulation results in Figures 4, 5. The scallop velum plays the role of the check valve function and empowers the scallops to steer. We design the robot artificial velum as fixed and movable parts to realize the two functions. According to the simulation results and conclusions in Figure 5A, we use the silicone sheet with appropriate stiffness to make the fixed part of the velum, such that it will bend inward under the pressure difference when the shells are opening and stay upright when the shells are closing. The scallop robot can thus realize the unidirectional control of the water flow, as shown in Figure 10B. When designing the movable part of the velum, according to the conclusion in Figures 7, 8, we designed a left-right tensile structure based on shape memory alloy spring (Park et al., 2020) (SMA spring) and torsion spring. When the robot needs to turn left, the left SMA spring in the movable part of the velum is electrified and contracted, pulling the flexible silicone sheet to reduce the size of the left jet aperture. Subsequently, the SMA is powered off and the flexible silicone sheet retracts the cavity under the drive of the torsion spring. The size of the two jet apertures is restored to the same and the robot moves in a straight line.

### 4.2 Motion control strategy

Flexible silicone sheets are driven by the SMA springs and torsion springs to form the jet apertures with different sizes. Under the opening and closing motions of the robot shells, asymmetric jets are formed and the robot realizes the steering motion accordingly. The turning radius of the robot can be modified by adjusting the size difference between the two jet apertures and the speed of the robot. The motion control strategy is described as follows. “A,” “B,” and “C” represent the left SMA spring, the right SMA spring, and the driving motor that drives the swing of the shells, respectively. We define the number “1” to indicate that the motor is in the driving state or the SMA spring is energized, and the number “0” to indicate that the motor is in the off state or the SMA spring is de energized.

**FIGURE 11:**
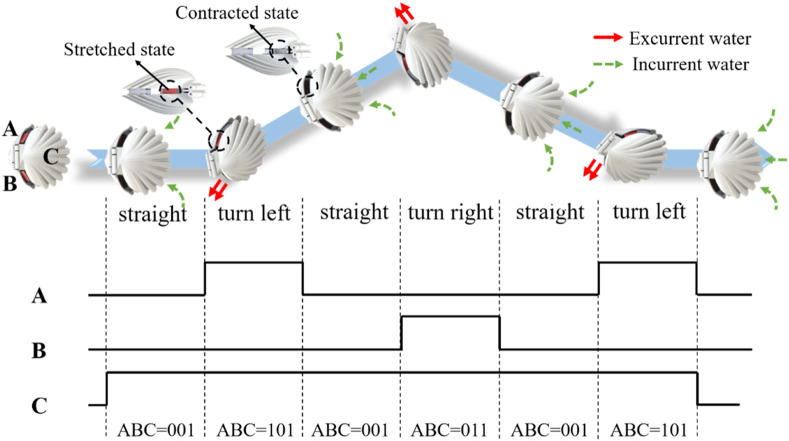
Sequential control strategy for robot steering. **(A,B)** represent the left and right SMA springs, respectively. **(C)** represents the driving motor that drives the swing of the shells.

As shown in Figure 11, when the robot needs to turn left, the corresponding control timing relationships are straight, turn left, straight, turn right, straight, and turn left. The action states of the left and right SMA springs and the driving motor are 001, 101, 001, 011, 001, and 101.

### 4.3 Experiments and results

**FIGURE 12:**
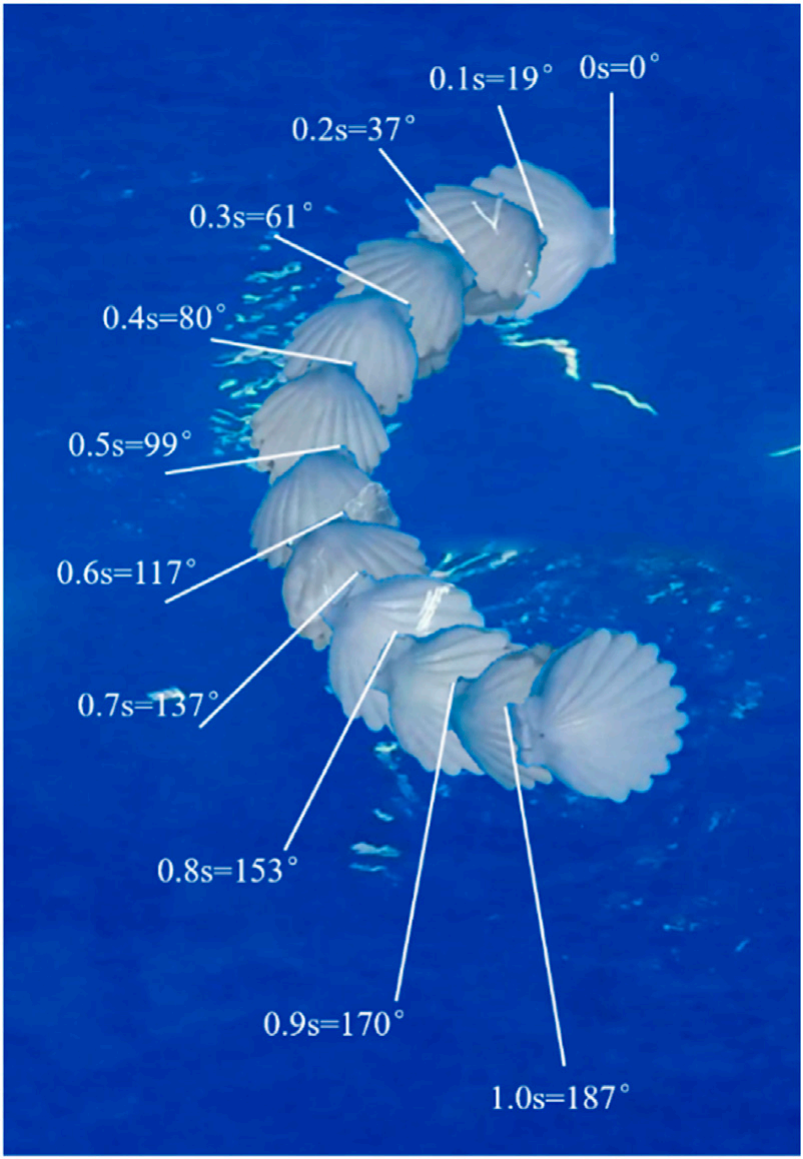
Motion trajectory of the robot.

We construct the experimental platform with length, width, and height of 318, 180, and 68 cm, respectively. As shown in the Figure 10C, motion cameras are installed above and in front of the platform to capture the movement of the robot. Given that the size of the experimental platform is considerably larger than that of the robot and the robot is programmed not to be near the edge of the experimental platform, the edge effect can be ignored in the experiments. The motion trajectory of the robot is shown in the Figure 12.

**FIGURE 13:**
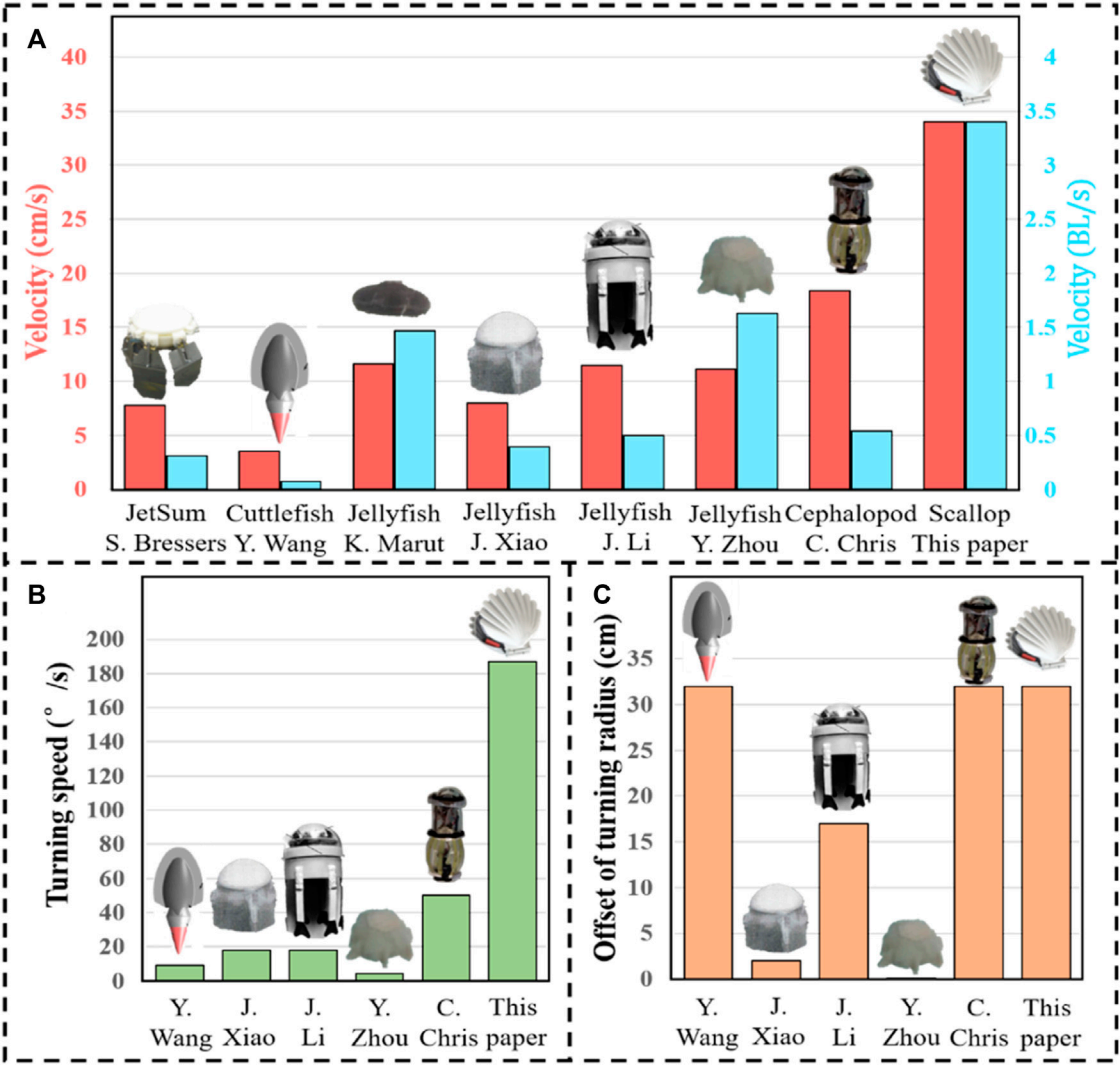
Propulsion performance comparison of bionic jet robots. **(A)** Average linear motion speed. **(B)** Turning speed. **(C)** Offset of turning radius.

Based on the motion recognition method for the scallop robot and decomposing the video into frames to measure the changes in the position and rotational angle of the scallop robot, the average linear motion speed and turning speed of the robot are obtained, which are compared with other high-mobility jet robaots, as shown in Table 1 and Figure 13. “Offset of turning radius” denotes the difference between the maximum turning radius of these jet robots and the turning radius of each robot.

**TABLE 1:** Propulsion performance comparison of bionic jet robots.

Research article	Name of the robot	Velocity (cm/s)	Velocity (BL/s)	Turning speed (°/s)	Offset of turning radius (cm)
Bressers et al., 2010	JetSum	6.99	0.28	Undisclosed	Undisclosed
Wang et al., 2018	Cuttlefish Robot	3.5	0.07	9 (undulating fin)	0 (undulating fin)
Marut et al., 2013	Jellyfish robot (iris mechanism)	11.6	1.47	Undisclosed	Undisclosed
Xiao et al., 2013	Jellyfish robot (6-bar linkage mechanisms)	7.9	0.38	9.2	Approx. 30
Li et al., 2016	Jellyfish robot (shape memory alloy)	11.5	0.5	18 (tentacle)	Approx.15 (tentacle)
Zhou et al., 2016	Jellyfish robot (soft modular structure)	11.1	1.63	16.3	31.95
Christianson et al., 2020	Cephalopod robot	18.4	0.54	50	0
This paper	Scallop robot	34	3.4	187	0

As shown in Figure 13A, the average linear motion speed of the scallop robot is significantly higher than that of the other jet robots, reaching 1.85 times of the sub-maximum value, and the body length speed reaches 2.09 times of the sub-maximum value. As shown in Figure 13B, in comparison with the existing high-mobility jet robots, the turning speed of the scallop robot reaches 3.74 times of the sub-maximum value. In Figure 13C, the bionic cuttlefish robot uses fin surface fluctuation, and the cephalopod inspired robot cannot actively adjust the jet direction; moreover, the difference in the turning radius of the scallop robot reaches 1.88 times of the second maximum value. In addition, the scallop robot can achieve a rapid *in situ* steering of 130°/s.

## 5 Conclusion remarks and future works

In this work, we have established the computational models of shells to explore the influence of different shapes, grooves, and cambers of scallop shells on the propulsion force during the swing process. It is proved that the camber-grooved have a great drag reduction effect on the forward motion. The shell structure of the scallop robot is designed accordingly. The computational model of the scallop is established, and the pressure distribution of the scallop’s inner and outer surfaces is obtained. On this basis, the power of the driving motor and the fixed part of the scallop robot’s artificial velum were designed. The influence of the position of the jet apertures on the movement is explored, and the effect of the jet propulsion and the swing shells of the scallop on its movement is preliminarily decoupled. We find that the scallop can adjust the jet propulsion force by regulating only the velum and accordingly design the movable part of the scallop robot’s artificial velum. Finally, the developed scallop robot possesses a high steering performance, and the experimental results show that the scallop robot can achieve fast steering of 187°/s and *in situ* steering of 130°/s. On the basis of stable swimming performance and good swimming mobility, the scallop robot can provide us a new propulsion mechanism in underwater bionic robots.

Although the reduction in drag due to the camber-grooved structure for scallop locomotion has been preliminarily demonstrated, quantitative analysis of the influence of groove quantity, groove shape, and arch curvature on scallop propulsion effectiveness is not provided. Furthermore, there is a lack of in-depth research on the motion process of biological scallops, particularly regarding the duration of different stages and the length of the motion cycle in various species of scallops during their locomotion.

Future work primarily involves further exploring the drag reduction effects of different shell morphologies and determining the optimal time ratios of the three stages during the locomotion process for different species of scallops. This would provide a stronger basis for selecting the appropriate time parameters in our design and potentially uncover additional insights for the evolution of scallop biomechanics. Besides, in future works, the mathematical model of steering motion will be established, enabling the robot to realize higher maneuverability, such as flipping. The motion efficiency of the scallop robot will be calculated and compared with other jet robots. Moreover, we will focus on installing more sensors on the robot to apply it in underwater exploration and surveillance tasks.

## Data Availability

The raw data supporting the conclusion of this article will be made available by the authors, without undue reservation.
