# Supplementary material for: Optimal design and development of a fast steering robot inspired by scallops
Source: Front Bioeng Biotechnol. 2024 Jan 8;11:1297727. doi: 10.3389/fbioe.2023.1297727 (PMC10800569; doi:10.3389/fbioe.2023.1297727)
Supplement: Supplementary file 2 [file DataSheet1.docx]

*1.The calculation of the force term (F) in the Navier-Stokes equation:*

The simulation is proceeded via fluid simulation software FLUENT. The semi-implicit method for pressure-linked equation (SIMPLE algorithm) is used to achieve the pressure-velocity coupling. Here, let the each volume of each control volume be $dV$. Calculate the mass of fluid in each control volume:

$dm=\rho dV$ (1)

where *ρ* is the fluid density and *m* is the mass.

Distribute external forces to each control volume according to mass. Set the body force distribution *f(x,y,z)* acting on the fluid. For each control volume, calculate the body force:

$dF=f(x,y,z)dm=f(x,y,z)\rho dV$ (2)

Summing up the body forces acting on the fluid in the entire computational domain, the total body force *F* can be derived as:

$F=\int\Omega f(x,y,z)\rho dV$ (3)

where *Ω* is the entire computational domain.

*2. The mathematical background behind the calculation of forces in Figure 3:*

The forces in Fig. 3 are the thrusts along the Y direction for each case. The pressure force is caused due to water viscosity. The components of the forces acting on the shell, $F_{x}$, $F_{y}$, $F_{z}$ can be evaluated by integrating the projection of the pressure and shear stress in the X, Y, and Z directions, respectively. The total component along the X direction is computed by adding the component X of the pressure and viscous forces acting on the shell as follows:

$F_{y}=F_{py}+F_{vy}$ (4)

where $F_{py}$ is the pressure force, and $F_{vy}$ is the viscous force in the Y direction.

The pressure force along the Y direction is expressed as:

$F_{py}=\int_{S} pn_{y}dS$ (5)

where *S* is the wetted surface area of the shell, *p* is the pressure force, $n_{y}$ is the unit vector in the Y direction.

The viscous force along the Y direction is expressed as:

$F_{vy}=\int_{S} \mu\frac{\partial u}{\partial y}dS$ (6)

where *μ* is dynamic viscosity, *u* is the velocity component and $\partial u/\partial y$ is the gradient of the velocity component with respect to the direction perpendicular to the wall.
